# The Northwest Passage opens for bowhead whales

**DOI:** 10.1098/rsbl.2011.0731

**Published:** 2011-09-21

**Authors:** Mads Peter Heide-Jørgensen, Kristin L. Laidre, Lori T. Quakenbush, John J. Citta

**Affiliations:** 1 Greenland Institute of Natural Resources, Nuuk, Greenland; 2 Polar Science Center, APL/University of Washington, Seattle, WA, USA; 3 Alaska Department of Fish and Game, Fairbanks, AK, USA

**Keywords:** bowhead whale, Northwest Passage, climate change, Arctic, sea ice

## Abstract

The loss of Arctic sea ice is predicted to open up the Northwest Passage, shortening shipping routes and facilitating the exchange of marine organisms between the Atlantic and the Pacific oceans. Here, we present the first observations of distribution overlap of bowhead whales (*Balaena mysticetus*) from the two oceans in the Northwest Passage, demonstrating this route is already connecting whales from two populations that have been assumed to be separated by sea ice. Previous satellite tracking has demonstrated that bowhead whales from West Greenland and Alaska enter the ice-infested channels of the Canadian High Arctic during summer. In August 2010, two bowhead whales from West Greenland and Alaska entered the Northwest Passage from opposite directions and spent approximately 10 days in the same area, documenting overlap between the two populations.

## 1. Introduction

The bowhead whale (*Balaena mysticetus*) played a major role in the initial exploration and exploitation of the Arctic, as humans pursued the species deep into ice-infested waters for oil, food and baleen (e.g. [1]). Inuit cultures and most notably the Thule culture depended on bowhead whales for subsistence for thousands of years [2]. Later, European and North American commercial whalers pursued bowhead whales throughout the Arctic.

**Figure 1.**
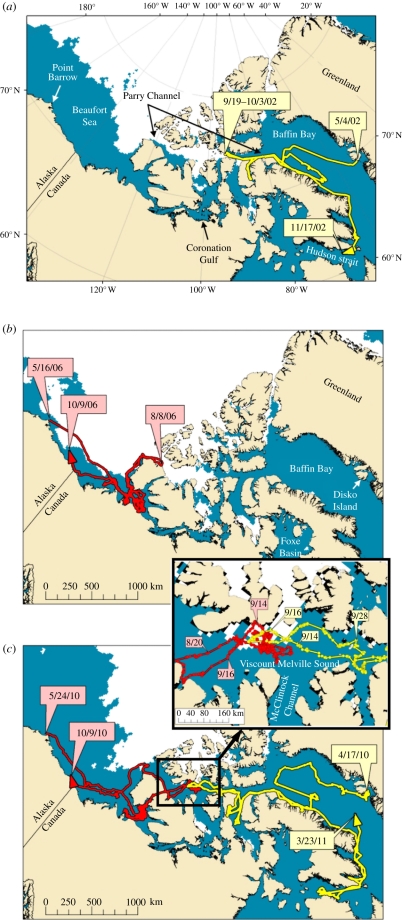
(*Opposite*.) The Northwest Passage with tracks of four bowhead whales and extent of sea ice with greater than 50% concentration (white fields). (*a*) Track of a whale tagged on 4 May 2002 in West Greenland and ice extent on 20 September 2002. (*b*) Track of a whale tagged in Alaska on 12 May 2006 and sea ice extent on 8 August 2006. (*c*) Track of a whale tagged on 24 May 2010 in Alaska, one tagged on 15 April 2010 in West Greenland, and sea ice extent on 14 September 2010. The insert shows the area where whales occurred together in 2010. The whale from Alaska was present in Viscount Melville Sound between 19 August and 18 September while the whale from Greenland was present from 11 to 28 September.

The Northwest Passage is a shipping pathway that connects the Atlantic and Pacific oceans through multiple small waterways in the Canadian High Arctic Archipelago. There are two routes: one begins in Baffin Bay and traverses Parry Channel into the Beaufort Sea, and the second branches south in Viscount Melville Sound and passes through Coronation Gulf (figure 1). Skeletal remains of bowhead whales on elevated beaches document that in the past bowhead whales occupied the Northwest Passage, although few remains exist from Coronation Gulf. These bones suggest that during the Early Holocene (approx. 11 000–8500 BP) there was sufficient open water in the interisland channels for bowhead whales to travel between the Atlantic and the Pacific oceans [3].

Arctic sea ice has been decreasing dramatically in extent and thickness since 1990 [4]. Model simulations indicate a continuing retreat and the possibility of ice-free summers in the Arctic Ocean within 20 years [5]. Bowhead whales summer in the high Arctic and winter in subarctic waters spending most of the year in the vicinity of sea ice. They are well adapted to ice-covered waters and can easily move through extensive areas of heavy sea ice cover [6], although to date it has been believed that the sea ice plug in the Northwest Passage was too concentrated to allow whales to pass through. Currently, five management stocks of bowhead whales are recognized: the Bering–Chukchi–Beaufort stock, the Hudson Bay–Foxe Basin stock, the Baffin Bay–Davis Strait stock, the Svalbard–Barents Sea stock and the Okhotsk Sea stock [7]. We used satellite telemetry to study the movements of whales in two of these stocks: the Bering–Chukchi–Beaufort (BCB) and the Baffin Bay–Davis Strait (BBDS) stock.

## 2. Material and methods

Bowhead whales have been instrumented with satellite transmitters near Disko Island in West Greenland since 2000 and in Alaska since 2006 [8,9]. Whales are pursued from small boats with outboard engines and tagging is conducted either by shooting a tag using a modified airgun or by placing the tag on the dorsal area of the whale with a 4–8 m long fibreglass pole [8,10]. Transmitter models have changed over time, but have primarily been Wildlife Computers SPOT5 or Splash tags (www.wildlifecomputers.com) modified for deployment on whales with a stainless spear with barbs that secure the tags in the blubber of the whales.

Positions of the whales were obtained through the Service Argos Collection and Location Service. Daily average positions were calculated including positions of both known precision (1, 2 and 3) and low quality (0, A and B) of unknown precision. Skin biopsies were used for a PCR-based method for the identification of sex [11]. Sea ice concentration was measured by the Advanced Microwave Scanning Radiometer—EOS (AMSR-E) sensor aboard the NASA's Aqua satellite (http://nsidc.org/index.html).

## 3. Results

Between 2001 and 2010, 122 bowhead whales were instrumented with satellite transmitters in spring in West Greenland, of which 44 transmitted through September each year (the month of greatest sea ice retreat in the Northwest Passage). Between 2006 and 2010, 58 bowhead whales were instrumented in Alaska and the western Canadian Arctic, of which 15 lasted through September (the latest date necessary to detect movements into the Northwest Passage before the autumn migration begins).

The first evidence that bowhead whales move at least partially into the Northwest Passage was obtained in 2002 when a 12 m long (sub-adult) bowhead whale tagged in West Greenland moved as far west as 93° W in the Northwest Passage in late September and early October (figure 1*a*). The whale ultimately returned east and headed out in Baffin Bay before moving south to winter in Hudson Strait. Four years later in 2006, a 14 m (adult) bowhead whale tagged near Point Barrow, Alaska was tracked north of Banks Island and along the coast into the Northwest Passage in early October reaching 800 km from the position of the whale tagged in West Greenland in 2002. In both 2002 and 2006, the Northwest Passage was blocked by dense sea ice that apparently prevented whales from penetrating the sea ice edge (figure 1*a*,*b*).

In 2010, two adult bowhead whales were tagged in spring, one in the BCB population and one in the BBDS population. The BCB whale was a 15 m male tagged near Point Barrow, Alaska, while the BBDS whale was a 17 m male and was tagged in West Greenland. In 2010, the Northwest Passage was largely free of sea ice by 10 August and the two whales moved into the Northwest Passage from opposite directions. Both whales were located in Viscount Melville Sound for more than two weeks in September, within 130 km of each other (less than 48 h travel for a bowhead whale). The whales crossed each other's paths in the Canadian Arctic Archipelago in Parry Channel in September 2010 before each returned to their normal seasonal range (figure 1*c*). It is not known what attracted the whales to this area, given the region has relatively low marine production in autumn compared with other known bowhead whale feeding areas [12].

## 4. Discussion

The movements of the four whales confirm previous observations that bowhead whales range widely and can traverse long distances during the open water season [9,13]. Bowhead whales from the BCB stock regularly undertake seasonal migrations between the Bering Sea and the western Canadian Arctic (a total annual distance of greater than 6000 km), while those from the BBDS stock migrate cross Baffin Bay and frequently move greater than 1000 km in less than a week [13]. The four whales that entered the Northwest Passage (including those from 2002 and 2006) were all males, perhaps indicating a more exploratory behaviour by males.

Sea ice in the Northwest Passage has been assumed to be a physical barrier separating bowhead whales from the Pacific and Atlantic oceans. Specifically, the thick sea ice in McClintock Channel has been suggested to act as a ‘plug’. Bowhead whales in this study travelled through the Northwest Passage using the Viscount Melville Sound and Parry Channel route, rather than the ‘plugged’ route through the McClintock Channel. It is likely that currents and tidal movements in summer in Viscount Melville Sound maintain small channels of open water large enough to be used by bowhead whales for passing through the Canadian High Arctic. The recent reduction of sea ice extent in August in the Arctic (8.2% per decade reduction between 1979 and 2006 in the Canadian Arctic; [5]) has probably facilitated greater access to the area and will ultimately allow for exchange through the Passage.

Assuming that less than 50 per cent ice coverage would allow bowhead whales to traverse the Northwest Passage, then monthly average sea ice concentration data (AMSR-E/Aqua satellite) between 1979 and 2010 demonstrate that Parry Channel was open to passage by bowhead whales during September in 1998, 1999, 2007, 2008 and 2010, and perhaps also in 1983. However, 50 per cent ice cover is a conservative estimate for the amount of open water needed for bowhead whales to travel through the Northwest Passage, as they can use small leads and cracks in the sea ice during migration.

During the commercial whaling period (i.e. pre-1900), several harpoon heads of Atlantic origin were discovered in bowhead whales harvested in the Chukchi Sea/western Arctic [14], but this information was largely dismissed as anecdotal by scientists. Recent genetic studies compared DNA of whales from Foxe Basin, Canada to whales from Alaska and suggest genetic mixing, although results are based on a small sample size from a highly segregated population [15,16]. The lack of genetic differentiation between whales in the Pacific and the Atlantic, acknowledging that samples are taken several thousand years apart, suggests that some exchange of individuals occurred between whales in Svalbard and Alaska [17]. The genetic similarity and satellite tracking results presented here suggests that bowhead whales may persist in a metapopulation structure with periodic exchanges between subpopulations depending on sea ice conditions. Given recent rates of sea ice loss, climate change may eliminate geographical divisions between stocks of bowhead whales and open new areas that have not been inhabited by bowhead whales for millennia (e.g. North of Greenland and north of the Canadian Archipelago; [18,19]).

The documented movements of bowhead whales in the Northwest Passage are perhaps an early sign that other marine organisms have begun exchanges between the Pacific and the Atlantic Oceans across the Arctic [20]. Some of these exchanges may be harder to detect than bowhead whales, but the ecological impacts could be more significant should the ice-free Arctic become a dispersal corridor between the two oceans.
